# Amorphous System of Hesperetin and Piperine—Improvement of Apparent Solubility, Permeability, and Biological Activities

**DOI:** 10.3390/ijms24054859

**Published:** 2023-03-02

**Authors:** Kamil Wdowiak, Andrzej Miklaszewski, Robert Pietrzak, Judyta Cielecka-Piontek

**Affiliations:** 1 Department of Pharmacognosy, Faculty of Pharmacy, Poznan University of Medical Sciences, Rokietnicka 3, 60-806 Poznan, Poland; 2 Institute of Materials Science and Engineering, Poznan University of Technology, Jana Pawla II 24, 61-138 Poznan, Poland; 3 Faculty of Chemistry, Adam Mickiewicz University in Poznań, Uniwersytetu Poznańskiego 8, 61-614 Poznan, Poland

**Keywords:** amorphous dispersions, drug delivery, hesperetin, piperine

## Abstract

The low bioaccessibility of hesperetin and piperine hampers their application as therapeutic agents. Piperine has the ability to improve the bioavailability of many compounds when co-administered. The aim of this paper was to prepare and characterize the amorphous dispersions of hesperetin and piperine, which could help to improve solubility and boost the bioavailability of both plant-origin active compounds. The amorphous systems were successfully obtained by means of ball milling, as confirmed by XRPD and DSC studies. What’s more, the FT-IR-ATR study was used to investigate the presence of intermolecular interactions between the systems’ components. Amorphization enhanced the dissolution rate as a supersaturation state was reached, as well as improving the apparent solubility of both compounds by 245-fold and 183-fold, respectively, for hesperetin and piperine. In the in vitro permeability studies simulating gastrointestinal tract and blood-brain barrier permeabilities, these increased by 775-fold and 257-fold for hesperetin, whereas they were 68-fold and 66-fold for piperine in the GIT and BBB PAMPA models, respectively. Enhanced solubility had an advantageous impact on antioxidant as well as anti-butyrylcholinesterase activities—the best system inhibited 90.62 ± 0.58% of DPPH radicals and 87.57 ± 1.02% butyrylcholinesterase activity. To sum up, amorphization considerably improved the dissolution rate, apparent solubility, permeability, and biological activities of hesperetin and piperine.

## 1. Introduction

Poor bioaccessibility and solubility are important factors in limiting the overall bioavailability of many compounds, including active substances derived from plants. Furthermore, in the case of polyphenolic natural compounds, intestinal protein efflux and cytochrome P450 metabolism are crucial factors to consider [1]. Hesperetin is a plant-derived compound that shows significant potential for preventing and supporting the treatment of chronic diseases. Its antioxidant, anti-inflammatory, anti-diabetic, anti-cancer, and neuroprotective activities have been well documented [2,3,4,5,6,7]. Hesperetin is found in substantial amounts in citrus fruits such as sweet oranges (*Citrus sinensis* L.) Osbeck and lemons (*Citrus limon* L.) Burm [2]. However, its role as a preventive or therapeutic agent is limited by its poor solubility, which translates into limited bioavailability. To date, several methods to improve hesperetin solubility have been described. Stahr et al prepared nanocrystals of hesperetin, which resulted in an enhancement in the dissolution rate as well as apparent solubility [8]. They observed a rapid increase in apparent solubility just after the start of the dissolution process, the so-called “spring effect,” without the parachute phenomenon, meaning that there was a high degree of increase in solubility at the beginning and a decrease in the amount of dissolved hesperetin over time [8]. Next, Wang et al fabricated self-assembling rebaudioside A nanomicelles with hesperetin, which considerably increased solubility and provided a sustained release. The systems were also characterized by enhanced biological potential with regard to anticancer activity [9]. While Gu et al produced hesperetin micelles with D-α-tocopheryl polyethylene glycol succinate and phosphatidylcholine that increased solubility by 21.5-fold, boosted antioxidant activity, and enhanced bioavailability by about 16.2-fold, whereas phosphatidylcholine enhanced solubility, antioxidant potential as well as bioavailability by 20.7-fold, 3.9-fold and 18-fold, respectively [10]. Trendafilova et al developed systems of hesperetin with Mg- and Ag-modified SBA-16 carriers, which translated into a higher apparent solubility and dissolution rate [11].

Piperine is an alkaloid of natural origin found in black pepper (*Piper nigrum* L.). It exhibits a number of health-promoting properties, such as anti-inflammatory, anti-diabetic, anti-cancer, and neuroprotective [12,13,14,15]. It is characterized by poor solubility, so researchers have tried a number of approaches to fight this issue. Zafar et al fabricated a self-nanoemulsifying drug delivery system with piperine. This approach improved its dissolution profile and permeability. It also contributed to better bioavailability in vivo as well as therapeutic efficacy such as anti-hypertensive, antibacterial, and antioxidant activities [16]. In turn, Zaini et al prepared piperine-succinic acid cocrystals, which were characterized by improved solubility and dissolution rate [17]. Ren et al developed piperine-loaded nanoparticles that exhibited enhanced dissolution rate, oral bioavailability, and brain delivery. Moreover, these nanoparticles showed great anti-epileptic activity [18]. Imam et al used solvent evaporation and microwave irradiation to create binary and ternary complexes of piperine with hydroxypropyl-β-cyclodextrin and D-α-tocopheryl polyethylene glycol succinate. Ternary complexes considerably improved piperine solubility and dissolution rate [19].

Amorphization is one of the promising techniques for improving the solubility of active compounds. Disruption of the crystalline structure and obtaining an amorphous state significantly improve the solubility and dissolution rate of compounds [20,21]. Taking into account the potential benefits of the combination of hesperetin with piperine and the limitations in the application of both compounds resulting from their poor solubility, the subject of this study was the development of an amorphous system containing hesperetin and piperine. Combining hesperetin with piperine could be beneficial in terms of bioavailability since the co-administration of poorly soluble compounds and bioenhancing substances, like piperine, is supposed to boost the bioavailability. A similar idea was proposed by Liu et al.; however, their approach involved obtaining hesperetin-piperine co-crystals, and as a result of the improved dissolution of both tested compounds after their introduction into the crystals, their concentration in the blood was increased [22].

The goal of this study was to create amorphous hesperetin-piperine dispersions with improved solubility for both compounds. The following stages of the research included (1) amorphous system preparation via ball milling, (2) amorphous system identification and characterization of physicochemical properties of its components, such as dissolution rate and permeability, and (3) evaluation of biological activity—antioxidant and neuroprotective activities. 

## 2. Results

The systems were prepared by ball milling at various mass ratios using a vinylpyrrolidone-vinyl acetate copolymer in a 6:4 ratio (known as Kollidon VA64 or PVP VA64) as the carrier to prevent crystallization during the dissolution process and to ensure supersaturation was maintained. After confirming the amorphization, tests were carried out to characterize the physicochemical properties such as dissolution rate, solubility, and permeability. Then, it was checked whether the amorphization had an impact on the biological potential of the systems.

### 2.1. Solid-State Identification

#### 2.1.1. X-ray Powder Diffraction (XRPD)

**Figure 1:**
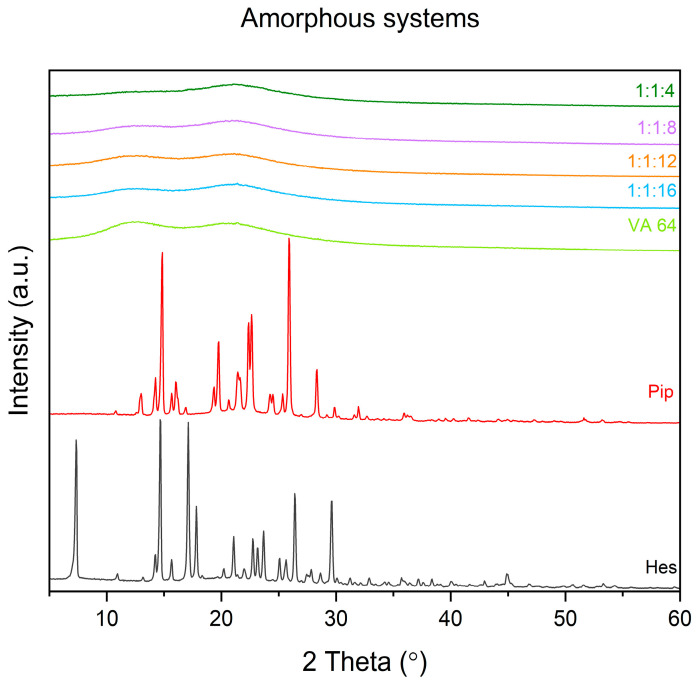
Diffractograms of raw compounds and amorphous systems. Amorphous systems were named as a mass ratio of individual components, Hes:Pip:VA 64 (Hes: hesperetin, Pip: piperine, and VA 64: PVP VA 64).

XRPD analysis gave some information on changes in solid-state form due to the milling process (Figure 1). In the case of unmodified compounds, the diffractograms are characterized by well-defined sharp peaks, thus indicating their crystalline nature. For hesperetin, peaks are seen at 7.33, 14.23, 14.64, 15.66, 17.10, 17.82, 20.22, 21.08, 21.99, 22.72, 23.16, 23.68, 25.07, 25.64, 26.39, 27.82, 28.65, 29.63° 2 Theta whereas for piperine they are visible at 13.01, 14.25, 14.84, 15.66, 16.02, 16.87, 19.35, 19.76, 20.65, 21.43, 22.37, 22.63, 24.25, 24.50, 25.34, 25.89, 28.34, 29.88, 31.96° 2 Theta. In the XRPD pattern of the obtained systems, a “halo effect” appeared, indicating that they are in an amorphous state. XRPD analysis of physical mixtures shows a pattern characteristic for amorphous materials and noticeable peaks suggesting the presence of crystalline material (Supplementary Materials Figure S1).

#### 2.1.2. Differential Scanning Calorimetry (DSC)

**Figure 2:**
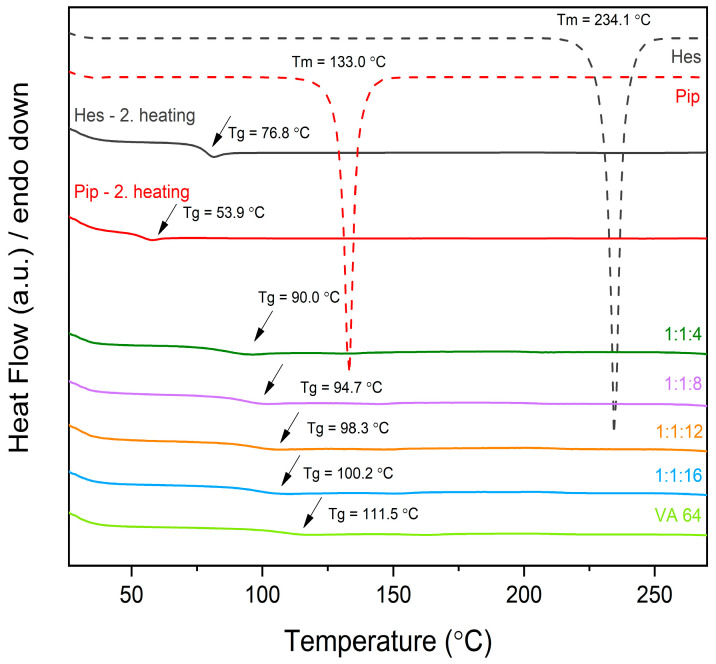
Thermograms of raw compounds and amorphous systems. Amorphous systems were named as a mass ratio of individual components, Hes:Pip:VA 64 (Hes: hesperetin, Pip: piperine, and VA 64: PVP VA 64).

To further confirm the XRPD study results as well as observe phase transitions, the DSC study was performed. Hesperetin in the first heating run shows a sharp endothermic peak at 234.1 °C, whereas piperine is at 133.0 °C. These thermal events correspond to the melting points of each compound. In the second heating of raw compounds, one can notice glass transitions of amorphous form, Tg = 76.8 °C and Tg = 53.9 °C for hesperetin and piperine, respectively. In the DSC patterns of amorphous systems, there is a lack of endothermic peaks corresponding to the melting points of individual components, which confirms the amorphization (Figure 2). Moreover, the single-glass transition is noticeable, suggesting good miscibility and molecular dispersion of components in amorphous systems. It’s worth noting that as the amount of polymer in a system increases, the glass transition shifts to higher values, which could indicate that the polymer provides better molecular mobility restriction.

#### 2.1.3. Fourier Transform Infrared Spectroscopy with Attenuated Total Reflectance (FTIR-ATR)

To investigate the presence of intermolecular interactions in the systems, FT-IR analysis was performed. Changes in FT-IR spectra, such as shift, disappearance, and reduction in intensity, as well as the appearance of new peaks, are strong indicators of interaction formation.

Each component of the system has various possible sites for hydrogen bond formation. Hesperetin is rich in hydroxyl groups, meaning it will most likely participate in hydrogen bonding as a proton donor. Piperine possesses a potent hydrogen bond acceptor, which is the oxygen atom of the amide group, as well as a proton donor, i.e., hydrogen, in the methylendioxyphenyl group [23,24]. Kollidon VA 64 has two hydrogen bond acceptor groups, which are the carbonyl group and vinyl acetate [25].

**Figure 3:**
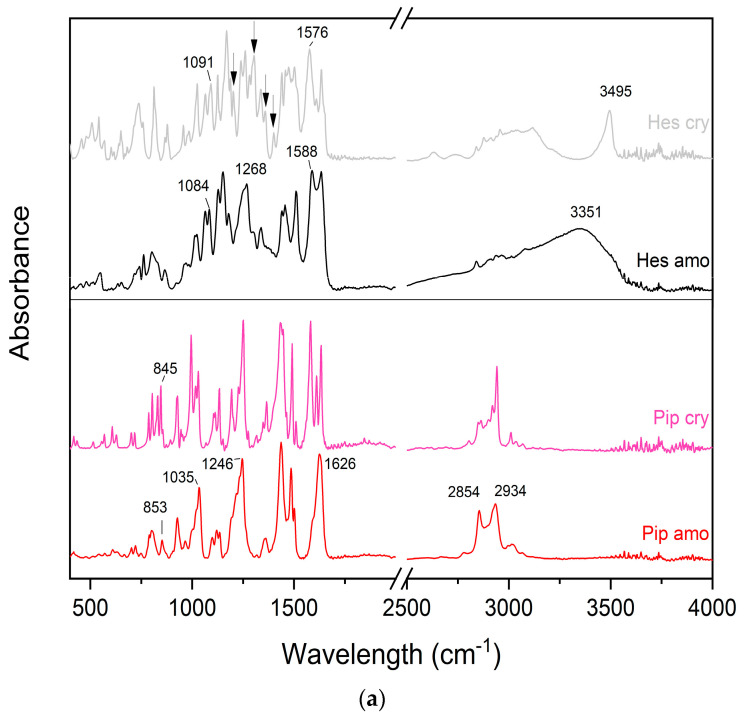

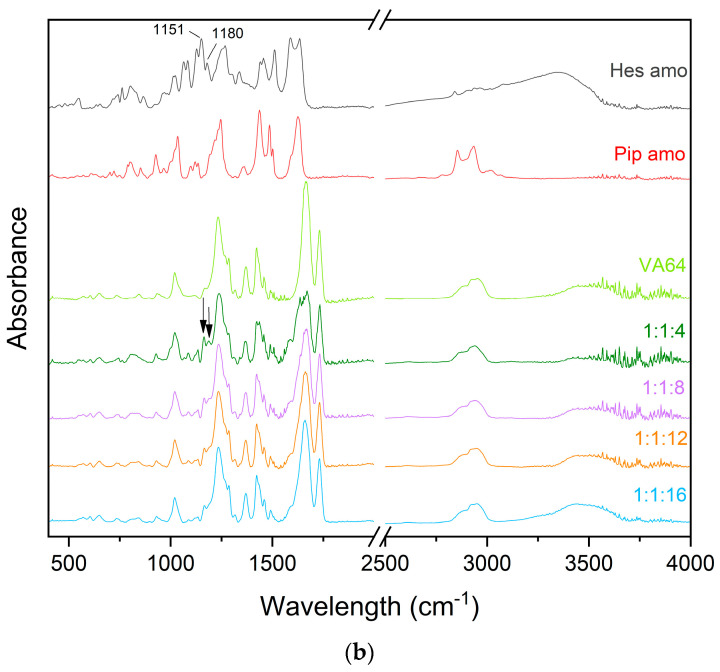
FTIR-ATR spectra of raw hesperetin and piperine in crystalline and amorphous forms (**a**), as well as amorphous raw compounds, Kollidon VA 64, and amorphous systems (**b**). Amorphous systems were named as a mass ratio of individual components, Hes:Pip:VA 64 (Hes: hesperetin, Pip: piperine, and VA 64: PVP VA 64). Black arrows indicate changes.

When comparing raw compounds in crystalline and amorphous forms, clear changes in FT-IR spectra are evident (Figure 3a). In the case of hesperetin, the crystalline form shows a sharp peak at 3495 cm^−1^ and a broad peak in the range 2839–3190 cm^−1^, which, in the case of the amorphous form, merged into a single peak with a broad base and an absorption maximum at 3351 cm^−1^. In addition, the peaks at 1260 and 1240 cm^−1^ merged to give a peak at 1268 cm^−1^. A shift of the peak at 1576 to 1588 cm^−1^ and the peak at 1091 to 1084 cm^−1^ can also be seen. Moreover, the peaks at 1611, 1402, 1359, 1305, and 1203 cm^−1^ disappeared and are not visible in the spectrum of the amorphous form of hesperetin. In the 900–400 cm^−1^ range, a significant reduction in intensity and broadening of the peaks can be seen. As for piperine, the broad peak with several maxima at 2942, 2919, 2863, and 2849 cm^−1^ merged into one broad peak with two absorption maxima at 2934 and 2854 cm^−1^. The peak with three distinct maxima at 1634, 1609, and 1582 cm^−1^ merged into one, yielding a maximum at 1626 cm^−1^. The base of the peaks at 1512, 1490, and 1433 cm^−1^ broadened, causing these peaks to merge but retain their maxima. The peaks at 1249, 1227, and 1192 cm^−1^ merged into one, with the maximum at 1246 cm^−1^. The same is true of the peaks at 1029, 1018, and 994 cm^−1^; these merged into one with the maximum at 1035 cm^−1^. The peak at 845 shifted to 853 cm^−1^, the peak at 829 cm^−1^ disappeared, and the peak at 786 merged with the one at 802 cm^−1^. In the 750–400 cm^−1^ range, reductions in intensity and broadening of the bases of the peaks can be seen. The FT-IR/ATR spectra of raw compounds with main peaks description were placed in Supplementary Materials (Figure S2).

In the case of the spectra of the systems (Figure 3b), the individual bands can be assigned to the bands of the individual components of the systems, except for the bands at 1164 and 1187 cm^−1^, which may be hesperetin bands at 1150 and 1179 cm^−1^, respectively, which shifted in the systems. As the amount of polymer in the system increased, the bands originating from the polymer became dominant. In turn, the indicated shifts of the two peaks may suggest some interactions, but no changes are observed in the regions of special interest, i.e., at the bands of functional groups that may be involved in hydrogen bond formation, therefore they cannot be clearly confirmed with a high degree of certainty.

### 2.2. Physicochemical Characterization of Amorphous Systems

#### 2.2.1. Dissolution Rate Studies

**Figure 4:**
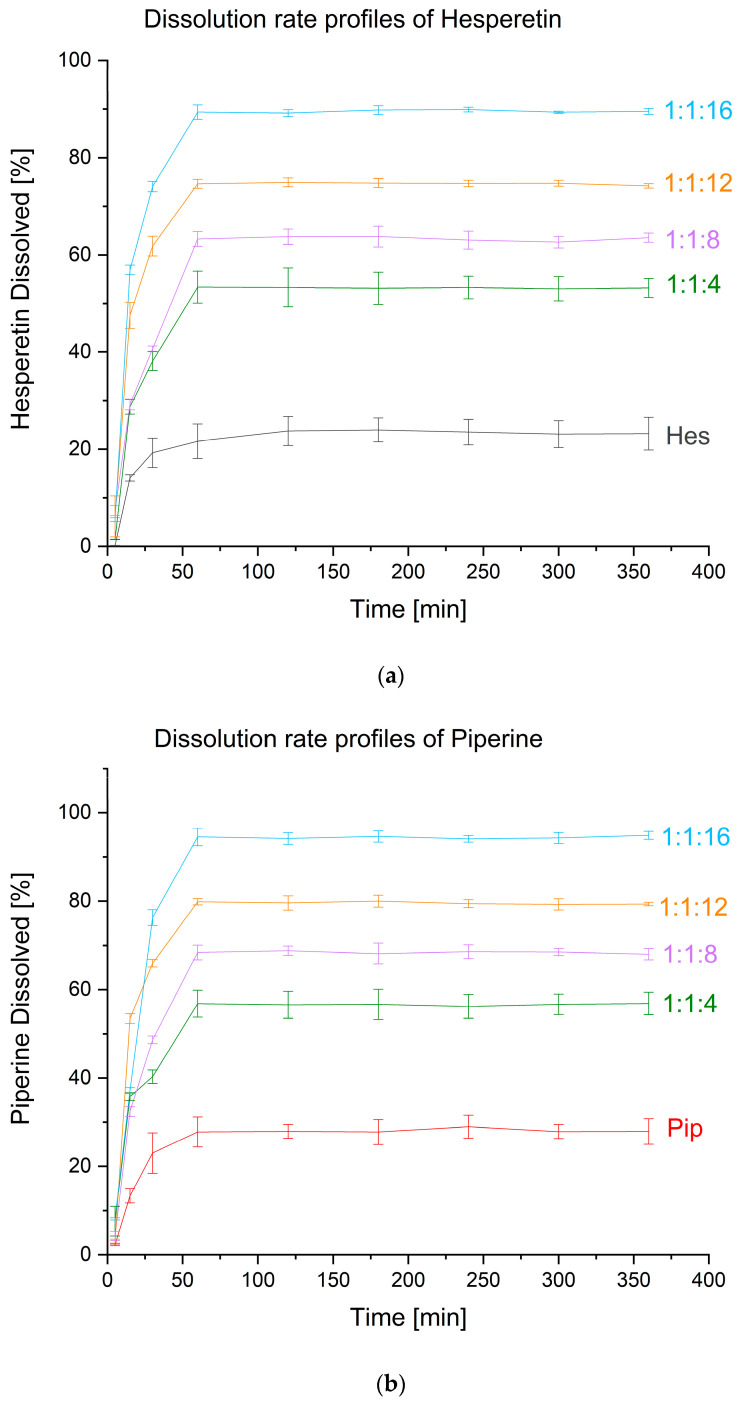
Dissolution rate profiles for amorphous systems of hesperetin (**a**) and piperine (**b**). Amorphous systems were named as a mass ratio of individual components, Hes:Pip:VA 64 (Hes: hesperetin, Pip: piperine, and VA 64: PVP VA 64).

One of the advantages of amorphous dispersions is that they improve the dissolution rate profile. The dissolution profiles for both substances showed an increase in the level of apparent solubility compared to raw compounds, so the obtained amorphous dispersions ensured that a supersaturated state was obtained. In both cases, the amorphous systems reached the plateau state after about an hour, and it was maintained until the end of the test, i.e., 6 h. In the case of raw hesperetin, 23.75 ± 2.99% dissolved after one hour, while raw piperine reached the level of 27.79 ± 3.38% of the dose added to the dissolution medium (Figure 4). As for the amorphous systems, the best improvement in apparent solubility was provided by the hesperetin-piperine-VA64 system in a mass ratio of 1:1:16. It allowed the dissolution of 89.39 ± 1.48% of hesperetin and 94.51 ± 1.97% of piperine.

#### 2.2.2. Solubility Study

Solubility studies were performed in phosphate buffers at pH 6.8. The solubility of raw compounds was determined to be as low as 0.005 ± 0.001 mg/mL and 0.006 ± 0.001 mg/mL for hesperetin and piperine, respectively. Solubility was significantly improved by using systems. The best amorphous systems turned out to be hesperetin-piperine-VA64 1:1:16. The compounds in this system reached concentrations above 1 mg/mL, giving 245-fold and 183-fold better solubility, with regards to raw compounds, for hesperetin and piperine, respectively. The results of the solubility studies are presented in Table 1.

#### 2.2.3. Permeability Studies

**Figure 5:**
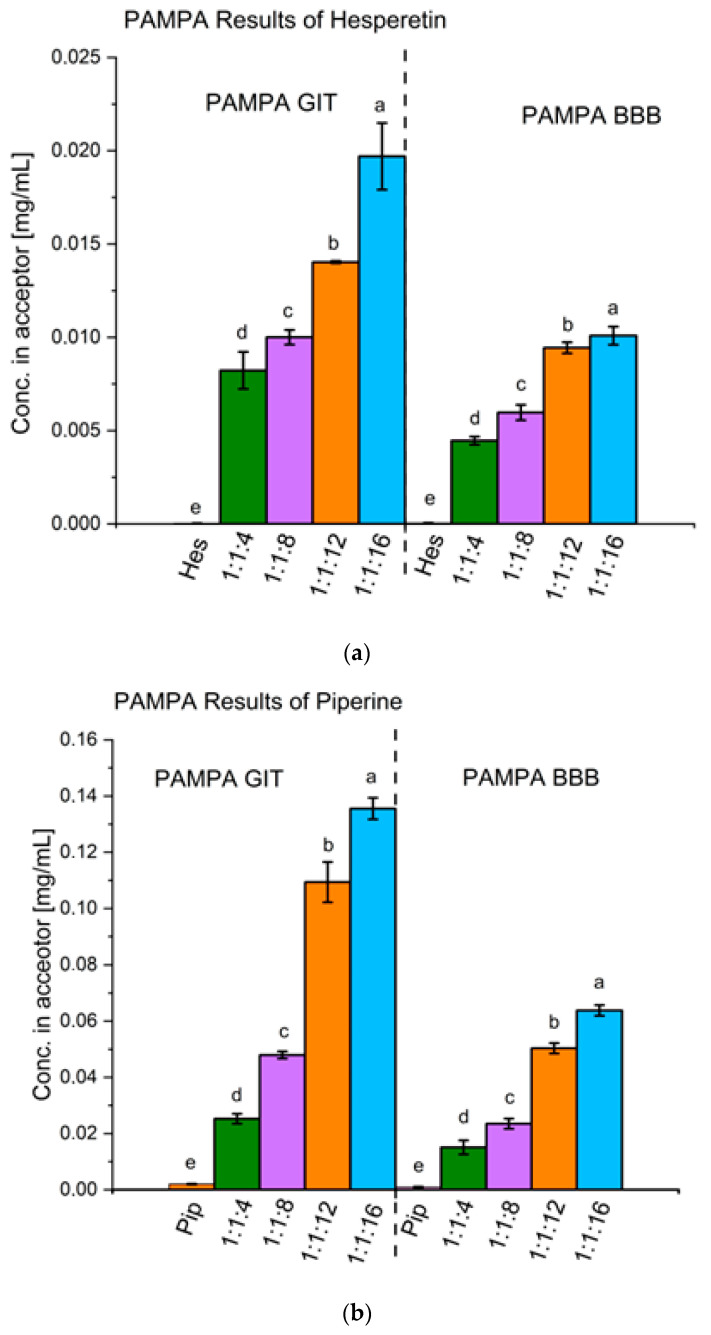
The results of the in vitro permeability assay for hesperetin (**a**) and piperine (**b**). Amorphous systems were named as a mass ratio of individual components, Hes:Pip:VA 64 (Hes: hesperetin, Pip: piperine, and VA 64: PVP VA 64). The statistically significant values are presented as a–e, with “a” being the highest value (*p* < 0.05).

In order to determine whether the obtained systems led to improved permeability, the parallel artificial membrane permeability assay (PAMPA) model was used to simulate passive diffusion through the cell walls of the gastrointestinal tract (GIT) and across the blood-brain barrier (BBB). To test if the model compounds themselves tend to passively permeate through the aforementioned barriers, an apparent permeability coefficient was determined. For PAMPA GIT, it was determined to be 2.59 × 10^−6^ ± 2.37 × 10^−7^ cm/s and 3.73 × 10^−5^ ± 3.33 × 10^−6^ cm/s for hesperetin and piperine, respectively. Based on this, it can be concluded that the tested compounds have good permeability across the intestinal barrier. In the case of PAMPA BBB, the calculated coefficients are 7.05 × 10^−6^ ± 3.02 × 10^−6^ cm/s for hesperetin and 4.00 × 10^−5^ ± 1.89 × 10^−6^ cm/s for piperine, respectively, which means that these compounds will also be able to cross the blood-brain barrier. To compare the permeability of the compounds in systems, the concentrations obtained in the acceptor parts were set side by side. Here, one can see a clear improvement in permeability in both PAMPA models. The concentration of raw hesperetin was determined to be as low as 2.58 × 10^−5^ ± 6.16 × 10^−6^ mg/mL in the PAMPA GIT model and 3.89 × 10^−5^ ± 1.66 × 10^−5^ mg/mL in the PAMPA BBB model. The best system enhanced permeability, enabling a concentration of 2.00 × 10^−2^ ± 2.00 × 10^−3^ mg/mL in the GIT model and 1.01 × 10^−2^ ± 5.02 × 10^−4^ mg/mL in the BBB model, meaning that these parameters increased by 775-fold and 257-fold, respectively. Similar observations can be reported for piperine. The concentration in the acceptor part increased from 2.01 × 10^−3^ ± 1.00 × 10^−4^ mg/mL to 1.36 × 10^−1^ ± 4.07 × 10^−3^ mg/mL in the GIT model and from 1.03 × 10^−3^ ± 1.04 × 10^−4^ mg/mL to 6.41 × 10^−2^ ± 2.00 × 10^−3^ mg/mL in the BBB model, which translated into an increase of 68- and 66-fold, respectively. The results of permeability studies (Figure 5) are related to what was observed in the solubility study. Both hesperetin and piperine are well permeable through biological barriers; therefore, their bioavailability is limited by poor solubility.

### 2.3. Biological Activity Studies

This biological activity research looked at the antioxidant (2, 2-diphenyl-1-picrylhydrazyl radical—DPPH radical) and butyrylcholinesterase (BChE) suppressing abilities. It is noticeable that the amorphization process and, as a consequence, improved solubility had an advantageous impact on biological activity. The best activity was shown by the Hes:Pip:VA 64 system at a mass ratio of 1:1:16, which reduced the DPPH radical at 90.62 ± 0.58% and suppressed BChE activity at 87.53 ± 1.02%, whereas a physical mixture of raw compounds in a 1:1 mass ratio produced 2.51 ± 1.05% and 2.12 ± 0.53% of inhibition in the DPPH and BChE tests, respectively (Table 2).

## 3. Discussion

Hesperetin and piperine are plant origin active substances that have a vast pro-healthy potential when it comes to prophylactic as well as the treatment of various diseases. Both compounds possess well-documented neuroprotective activity involving antioxidant and anti-neuroinflammatory activity as well as inhibition of toxic protein aggregation [14,26]. However, their use in therapeutic strategies is limited due to their low bioavailability, which is connected to insufficient levels of solubility. This in turn causes inadequate blood concentrations, which results in poor pharmacological effects. This work aims to develop amorphous systems to address these issues. When designing the dispersions, the authors took advantage of the beneficial effect of compounds on boosting bioavailability.

In our research, an environmentally friendly approach to preparing amorphous systems was used. The applied ball milling technique enabled the formation of amorphous systems. This observation was supported by the X-ray powder diffraction (XRPD) study, which revealed an amorphous pattern, as well as the differential scanning calorimetry (DSC) study, which showed a lack of crystalline structure in the systems due to the disappearance of the endothermic peaks corresponding to melting points and the appearance of the glass transition. What’s more, Fourier transform infrared spectroscopy with attenuated total reflectance (FT-IR-ATR) analysis made it possible to investigate intermolecular interactions. No obvious suggestions of the formation of hydrogen-bond-like interactions can be seen.

The amorphization process improved bioaccessibility, which means there are more free compounds available for absorption. This may influence permeability, as these two factors are closely related [27]. It is assumed that flavanone aglycones—hesperetin—have limited bioaccessibility but high intestinal permeability [27], meaning that the only limiting factor, in this case, is solubility. The same observation applies to piperine, since it also possesses high absorption potential. Hesperetin and piperine may be considered II-class compounds in the Biopharmaceutical Classification System. For most compounds, passive diffusion is the primary mode of absorption [28]. As amorphous systems were produced and the supersaturated state was maintained, it can be concluded that the Kollidon VA64 used in this study prevented crystallization of the model compounds. Apparent solubility, in the dissolution rate study, increased by 3.9-fold and 3.4-fold for hesperetin and piperine, respectively. In the case of amorphous dispersions, this is not obvious, as there is a high risk of precipitation of active substances due to reaching concentrations above their crystalline solubility. In addition, the rate of release of active ingredients from amorphous dispersions was controlled by the carrier. The tendency is that the more polymer there is in the system and, thus, in the dissolution medium, the greater the apparent solubility. Similar observations were reported by Szafraniec-Szczęsny et al. [29]. In their study on ezetimibe in amorphous dispersion with Kollidon VA64 obtained by spray drying, they pointed out the dependence of the amount of polymer in the system on the amount of dissolved active substance. They also included amorphous ezetimibe without a polymer carrier in the study. Despite the amorphous state, the achieved amounts of dissolved active ingredient were close to those of the crystalline substance, which also highlights that the presence of polymer is significant in the overall performance of the amorphous form in the dissolution process.

The supersaturation state achieved by amorphous systems can directly translate into improved absorption of active substances, as only free, dissolved molecules are capable of penetrating biological barriers. Several studies show that the supersaturated state contributes to achieving higher drug concentrations in the blood [30,31,32,33,34]. Accordingly, it can be expected that the amorphous dispersions obtained would also enable increased absorption of hesperetin and piperine. What’s more, since the solubilities of the compounds increased markedly, i.e., 245-fold for hesperetin and 183-fold for piperine, the potential for bioavailability enhancement is great.

It is also important that the systems provide a state of supersaturation within a 6-h window, i.e., the approximate time that intestinal contents can reside in the duodenum, the main site of absorption in the body [35]. The use of amorphous dispersion does not necessarily immediately imply that the aforementioned supersaturation will be stable over time. The behavior of the active substances will depend on the carrier and the extent to which it prevents crystallization. Knopp et al., using their celecoxib study as an example, showed that the performance of amorphous solid dispersion is significantly affected by crystallization [36]. They showed that the crystallization that occurs is directly related to the decrease in AUC. Moreover, this is strongly influenced by the polymer used as a carrier. In their study, the authors used HPMC and PVP, with PVP being more effective in preventing crystallization.

The prepared amorphous systems were characterized by significantly improved passive permeability compared to raw compounds, which was proven by the PAMPA model both in terms of absorption in the gastrointestinal tract and overcoming the blood-brain barrier. For hesperetin, the enhancement was up to 775-fold in the intestinal model and 257-fold in blood-brain barrier model studies, whereas, in the case of piperine, it was 68-fold in intestinal model studies and 66-fold in blood-brain barrier model studies. However, the bioavailability of these compounds is also closely related to active transport, which may hinder reaching the desired blood concentrations. It is worth noting that the BCRP (breast cancer resistance protein) transporter may be considered one of the biggest factors hampering hesperetin bioavailability since it contributes to the efflux of hesperetin [37]. The inhibition of this protein by piperine could considerably improve bioavailability. In fact, Bi Xiaoli et al supplied some pieces of evidence supporting this statement. They demonstrated that co-administration of piperine with silybin increased the bioavailability of silybin via the inhibition of the efflux transporters, including multidrug resistance-associated protein 2 (MRP2) as well as BCRP [38]. Moreover, Denni Yu et al showed that the co-administration of piperine with another compound boosts its bioavailability. The fabricated co-amorphous system of ursolic acid and piperine enhanced dissolution by about 7-fold as well as AUC values for both components in the pharmacokinetic study [39]. We assume that in the case of our ternary amorphous systems, similar observations could be anticipated. What’s more, as in the obtained systems, piperine is in an amorphous state, so one can expect it to show even greater potential in suppressing the activity of efflux proteins, which might result in a better capability to enhance permeability by this mechanism.

Increased bioaccessibility can improve hesperetin’s overall bioavailability. Omidfar et al obtained nanophytosmoes of hesperetin, which enabled them to reach higher blood concentrations in vivo than those of raw hesperetin. The authors attributed it to the amorphous nature of the systems as well as reduced particle size. Additionally, the factors contributing to better bioavailability may also be increased permeability and protection from first-pass metabolism provided by phytosomes [40]. In this context, our systems may ensure similar benefits since amorphization leads to improved solubility and stimulates passive diffusion, whereas piperine is expected to inhibit efflux as well as first-pass metabolism. All in all, these factors may lead to improved bioavailability of both active components of the ternary amorphous system. Moreover, one cannot forget that piperine, an alkaloid compound, also possesses great biological potential, and its bioaccessibility is limited by its solubility. Obtained amorphous systems may improve the bioaccessibility of both plant origin active ingredients, therefore boosting their extraordinary biological potential, which is hampered by poor solubility.

Neurodegenerative diseases seem to be a growing issue, especially in aging populations [41]. It is well documented that oxidative stress plays a crucial role in the development of neurodegenerative diseases. Oxidative stress is involved in the progression of neuroinflammation as well as the stimulation of protein misfolding and aggregation. In addition, it has an important role in damaging intracellular structures. The above mechanisms are factors that trigger cell death processes leading to neuronal loss [42,43]. Cholinesterases are important molecular targets to consider. Both hesperetin and piperine have been shown to have esterase enzyme inhibitory activity [44,45]. These enzymes take part in the regulation of acetylcholine levels and are thus engaged in cholinergic transmission [46]. What’s more, the presence of BChE has been confirmed in amyloid plaques as well as neurofibril tangles, which suggests involvement in Alzheimer’s disease pathophysiology [47,48]. It was demonstrated that BChE might transform β-amyloid plaques from a benign to a malignant form [48]. Furthermore, BChE appears to play an important role in the development of multiple sclerosis. A rise in its activity is associated with an increase in inflammation [49]. The development of amorphous ternary systems of hesperetin and piperine resulted in suppression of the DPPH radical up to 90.62 ± 0.58% as well as BChE activity to the extent of 87.53 ± 1.02%. These effects are probably related to the fact of improved solubility, since only free molecules could act on the DPPH radical or BChE enzyme and thus suppress their activity. In fact, Stahr et al prepared hesperetin nanocrystals, which showed potential in the treatment of Alzheimer’s disease [8]. In their study, higher apparent solubility was responsible for better anti-Alzheimer’s activity. This research supports the statement that enhanced solubility may contribute to increasing biological potential.

## 4. Materials and Methods

### 4.1. Materials

All materials including the tested compounds: hesperetin (purity > 95%) and piperine (purity > 95%, FG) were supplied by Sigma-Aldrich (Sigma-Aldrich, St. Louis, MO, USA), except for vinylpyrrolidone-vinyl acetate copolymer (Kollidon^®^VA64, PVP/VA, BASF, Ludwigshafen am Rhein, Germany), dimethyl sulfoxide (DMSO), sodium hydroxide (Avantor Performance Materials Poland S.A., Gliwice, Poland), acetic acid 98–100% (POCH, Gliwice, Poland), sodium dihydrogen phosphate (PanReac AppliChem ITW Reagents, Darmstadt, Germany), and methanol of an HPLC grade (J. T. Baker, Center Valley, PA, USA). High-quality pure water was prepared using a Direct-Q 3 UV purification system (Millipore, Molsheim, France; model Exil SA 67120). Prisma HT, GIT/BBB lipid solution, and acceptor sink buffer were supplied by Pion Inc. (Forest Row, East Sussex, UK).

### 4.2. Preparation of the Systems

The amorphous systems of Hes:Pip:VA 64 were obtained in different mass ratios, i.e., 1:1:4, 1:1:8, 1:1:12, and 1:1:16, by the means of ball milling (Retsch Mixer Mill MM 400). In short, 800 mg of the physical mixture was placed in a 25-mL stainless steel jar with 2 balls ø 10 mm, 3 balls ø 7 mm, and 3 balls ø 5 mm. The milling process was performed in six cycles, each lasting 20 min. The 5-min break was applied between cycles.

### 4.3. Solid-State Identification

#### 4.3.1. X-ray Powder Diffraction (XRPD)

The crystallographic structure of the samples were analyzed by an X-ray diffraction (XRD, Panalytical Empyrean, Almelo, Netherlands) equipment with the copper anode (CuKα—1.54 Å) at a Brag-Brentano reflection mode configuration with 45 kV and 40 mA parameters. The measurement parameters were set up for 3–60° with a 45-s step per 0.05° in all cases.

#### 4.3.2. Differential Scanning Calorimetry (DSC)

Thermal analysis was performed using a DSC 214 Polyma differential scanning calorimeter (Netzsch, Selb, Germany). Samples of about 5–8 mg were placed in crimped aluminum pans with a small hole in the lid. First, the samples were heated up to 80 °C and kept at this temperature for 8 min to remove water from the samples, then they were cooled down to 25 °C and heated again to 280 °C. To measure the glass transition value of raw compounds, they were heated up to 280 °C, then cooled down and heated again to 280 °C. The measurements were performed at a constant heating rate of 20 °C/min under a nitrogen atmosphere with a flow rate of 30 mL/min. The glass transition value was taken as a midpoint between on-set and end-point temperatures.

#### 4.3.3. Fourier Transform Infrared Spectroscopy with Attenuated Total Reflectance (FTIR-ATR)

The FTIR-ATR spectra were measured between 400 cm^−1^ and 4000 cm^−1^, with a resolution set to 1 cm^−1^, with a Shimadzu IRTracer-100 spectrometer equipped with a QATR-10 single bounce, a diamond extended range, and LabSolutions IR software (Warsaw, Poland). Amorphous forms of raw compounds were prepared by DSC by heating them to 280 °C and maintaining that temperature for 5 min.

### 4.4. Physicochemical Characterization

#### 4.4.1. HPLC Conditions

Concentrations of hesperetin and piperine during solubility, dissolution rate, and permeability studies were measured by high-performance liquid chromatography with the DAD detector (HPLC-DAD). In this study, a Shimadzu Nexera (Shimadzu Corp., Kyoto, Japan) equipped with an SCL-40 system controller, a DGU-403 degassing unit, a LC-40B XR solvent delivery module, a SIL-40C autosampler, a CTO-40C column oven, and a SPD-M40 photodiode array detector was used. For the stationary phase, a Dr. Maisch ReproSil-Pur Basic-C18 100 Å column with 5 µm particle size and 250 × 4.60 mm (Dr. Maisch, Ammerbuch-Entringen, Germany) was used. The mobile phase was methanol:0.1% acetic acid (80:20 *v*/*v*). The mobile phase was vacuum-filtered through a 0.45 µm nylon filter (Phenomenex, Torrance, CA, USA). The experimental conditions were as follows: 1.0 mL/min flow rate, wavelengths of 288 nm for hesperetin and 340 nm for piperine, and a column temperature of 30 °C. The injection volume differed depending on the assay. For the solubility study, it was 1 µL, whereas for the dissolution rate and permeability assays, it was 10 µL. The method’s duration was 10 min. The retention times were 4.11 min for hesperetin and 6.77 min for piperine. Chromatograms (Figure S3a,b) and validation parameters (Table S1) were placed in Supplementary Materials. 

#### 4.4.2. Media for Dissolution and Solubility Studies

Phosphate buffer at pH 6.8 was prepared according to the following description: in a 1000-mL volumetric flask, we placed 250 mL of 0.2 N potassium dihydrogen phosphate solution, then added 112 mL of 0.2 N sodium hydroxide solution, and filled the mixture up to 1000 mL with distilled water. High-quality pure water was prepared using a Direct-Q 3 UV purification system (Millipore, Molsheim, France, model Exil SA 67120).

#### 4.4.3. Dissolution Studies

The dissolution study was performed on the paddle apparatus. The amount of the compound and systems corresponding to 7.0 mg of each plant-origin active ingredient was added to the dissolution medium. The vessels were filled with 500 mL of phosphate buffer, pH 6.8; the temperature was maintained at 37 °C, and the paddles were set at a stirring speed of 50 rotations per minute. The 2.0 mL samples were withdrawn at predetermined time points with the replacement of equal volumes of temperature-equilibrated media.

#### 4.4.4. Solubility Studies

An excess amount of raw compounds and systems was placed in a 10 mL glass tube; then, 2.0 mL of phosphate buffer (pH 6.8) was added and left at room temperature for 3 h. The obtained solutions were diluted 1:1 with water, filtered through a 0.2 μm PTFE membrane filter (Sigma-Aldrich, St. Louis, MO, USA), and analyzed using the HPLC method.

#### 4.4.5. Permeability Studies

In vitro gastrointestinal (GIT) and blood-brain barrier (BBB) permeability were studied using the PAMPA (Parallel Artificial Membrane Permeability Assay) models. The sandwich consists of two 96-well microfilter plates. The PAMPA systems contain two chambers: the donor chamber at the bottom and the acceptor chamber at the top. The chambers are separated by a 120 μm thick microfilter disc coated with a 20% (*w*/*v*) dodecane solution of a lecithin mixture (Pion, Inc.). The donor solution was adjusted to pH ≈ 6.8 for GIT application and to pH ≈ 7.4 for BBB application using 0.5 M NaOH. The plates were combined and then incubated for 3 h for both models in a humidity-saturated atmosphere with the temperature set at 37 °C. To assess the apparent permeability coefficient factor (*Papp*), 5.0 mg of raw compounds were dissolved in 1.0 mL of DMSO. Then, we followed the manufacturer’s guidelines for further performance in the assay. The *Papp* factor was calculated according to the previously reported method [50]. In the case of the studied systems, the solutions were first prepared in the same manner as in the solubility study. Then the systems were diluted 1:1 with water, filtered through a 0.2 μm PTFE membrane filter, and further diluted 1:1 with DMSO. Next, the obtained solution was diluted 1:1 with donor solution for the GIT and BBB assays and placed in the donor compartment. The results were expressed as a concentration in the acceptor solution.

### 4.5. Biological Activities Studies

#### 4.5.1. Antioxidant Activity Assay

Briefly, 25.0 μL of studied solutions (prepared for concentration determination in a solubility study) were mixed with 175.0 μL of DPPH radical solution. The rest of the analysis was performed according to the outlined procedure [50].

#### 4.5.2. Determination of Butyrylcholinesterase (BuChE) Inhibition

The determination of BuChE inhibition was performed according to the previously reported method [50].

### 4.6. Statistical Analysis

Results are expressed as mean ± standard deviation. Statistical tests were performed using a one-way analysis of variance (ANOVA), and statistical differences using Duncan’s tests with a significance threshold of *p* < 0.05 were determined. All statistical analyses were performed using Statistica 13.1 software (TIBCO Software Inc., Palo Alto, CA, USA).

## 5. Conclusions

Obtaining amorphous systems of hesperetin and piperine seems to be the righteous approach to improving the overall bioavailability of both compounds.

We successfully obtained amorphous ternary dispersions of hesperetin and piperine. The systems were characterized by an improved dissolution rate, apparent solubility, permeability, and biological activities. Enhanced biological activity is strictly related to greater solubility, since only molecules that are freely dispersed in solution may act on biological targets. The administration of these systems might be beneficial in the context of various diseases due to the multidirectional mode of action of the studied plant-origin compounds.

## Figures and Tables

**Table 1:** The results of solubility studies of amorphous systems are presented as achieved concentrations and folds of improvement with regards to hesperetin and piperine.

System (Hes:Pip:VA 64)	Compound			
	Hesperetin		Piperine	
	Conc. [mg/mL]	Improv. [-fold]	Conc. [mg/mL]	Improv. [-fold]
Raw	0.005 ± 0.001 ^e^	-	0.006 ± 0.001 ^e^	-
1:1:4	0.269 ± 0.001 ^d^	58	0.167 ± 0.001 ^d^	27
1:1:8	0.312 ± 0.002 ^c^	67	0.237 ± 0.001 ^c^	38
1:1:12	0.509 ± 0.021 ^b^	110	0.674 ± 0.044 ^b^	109
1:1:16	1.136 ± 0.100 ^a^	245	1.134 ± 0.075 ^a^	183

The statistically significant values are presented as a–e, with “a” being the highest value (*p* < 0.05).

**Table 2:** The results of amorphous systems’ improved inhibition activities against DPPH radicals and BChE.

System (Hes:Pip:VA 64)	Assay	
	DPPH	BChE
	% of Inhibition	% of Inhibition
Raw	2.51 ± 1.05 ^e^	2.12 ± 0.53 ^e^
1:1:4	15.55 ± 1.15 ^d^	10.17 ± 1.24 ^d^
1:1:8	32.06 ± 1.22 ^c^	29.48 ± 1.87 ^c^
1:1:12	80.22 ± 0.54 ^b^	78.16 ± 2.89 ^b^
1:1:16	90.62 ± 0.58 ^a^	87.53 ± 1.02 ^a^

The statistically significant values are presented as a–e, with “a” being the highest value (*p* < 0.05).

## Data Availability

Data are available in a publicly accessible repository.

## Supplementary Materials

The supporting information can be downloaded at: https://www.mdpi.com/article/10.3390/ijms24054859/s1. References [51,52,53,54,55,56,57,58] were mentioned in Supplementary Materials.
