# Supplementary material for: Amorphous System of Hesperetin and Piperine—Improvement of Apparent Solubility, Permeability, and Biological Activities
Source: Int J Mol Sci. 2023 Mar 2;24(5):4859. doi: 10.3390/ijms24054859 (PMC10002548; doi:10.3390/ijms24054859)
Supplement: Supplementary file 1 [file ijms-24-04859-s001.zip › ijms-2221726-supplementary.pdf]

International Journal of *Molecular Sciences*

MDPI

Article

Amorphous System of Hesperetin and Piperine—Improvement of Apparent Solubility, Permeability, and Biological Activities

Kamil Wdowiak [1], Andrzej Miklaszewski [2], Robert Pietrzak [3] and Judyta Cielecka-Piontek [1,*]

[1] Department of Pharmacognosy, Faculty of Pharmacy, Poznan University of Medical Sciences, Rokietnicka 3, 60-806 Poznan, Poland
[2] Institute of Materials Science and Engineering, Poznan University of Technology, Jana Pawla II 24, 61-138 Poznan, Poland
[3] Faculty of Chemistry, Adam Mickiewicz University in Poznań, Uniwersytetu Poznańskiego 8, 61-614 Poznan, Poland
* Correspondence: jpiontek@ump.edu.pl

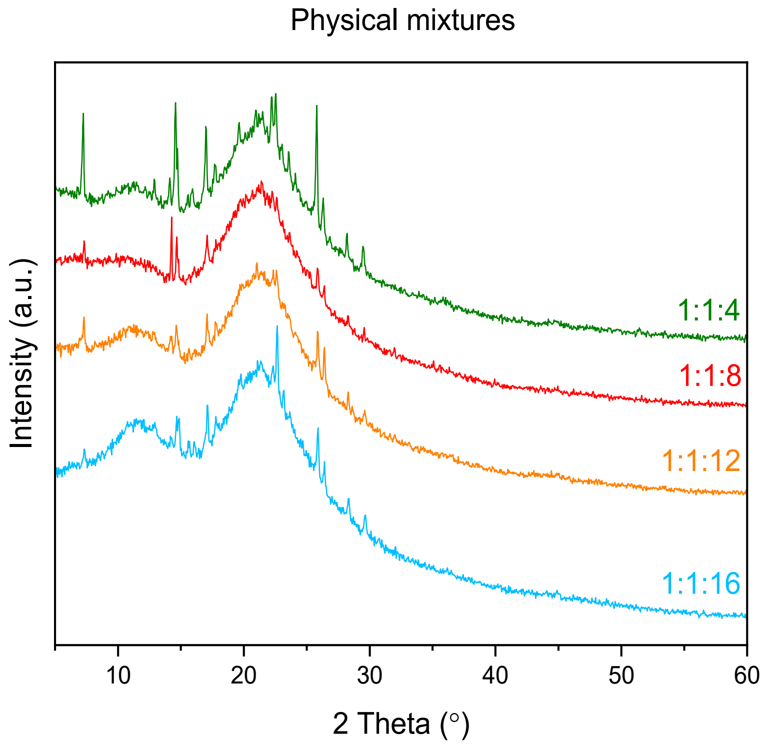

Figure S1. Diffractograms physical mixtures of the systems.

The FT-IR pattern of raw hesperetin is characterized by peaks such as 3499 cm-1 (O-H stretching), 1646 cm-1 (C=O stretching) strong bands at 1609-1294 cm-1 (C=C stretching of aromatic ring), 1465-1230 cm-1 (C-O stretching between the aromatic ring and hydroxyl/methoxy groups), 900-800 cm-1 (C-H rocking of aromatic ring) [51, 52]. Raw piperine showed characteristic peaks at 2939 cm−1 (C-H stretching), 1633 cm−1 (N-H bending), 1581 cm−1 (N-C=O stretching in carbonyl amide), 1506 cm-1 (C-NH asymmetric bending and C=C stretching of aromatic ring), 1437 cm-1 (C=CH2 CH2 deformation and bending), 1368 cm-1 (C-H bending), 1310 cm-1 (C-N asymmetric stretching), 1252 cm-1

(C-O stretching in methylenedioxy group), 927 cm-1 (C-O vibration in methylenedioxy-phenyl) [24,53,54,58]. The FT-IR spectra of pure Kollidon VA64 is described by the peak at 1730 cm−1 (vinyl acetate), 1668 cm−1 (C=O stretching) as well as 1290 cm−1 (C-N vibration) [55-57].

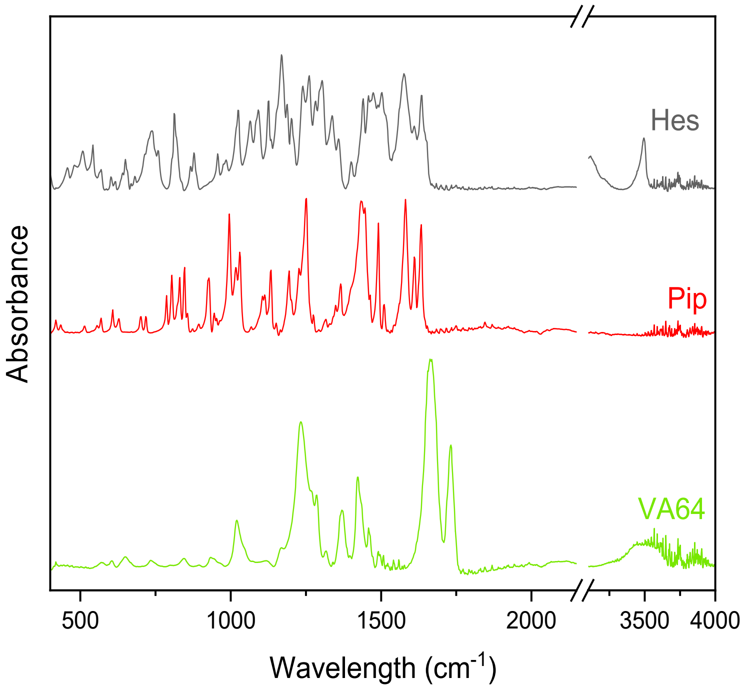

Figure S2. FT-IR/ATR spectra of raw compounds

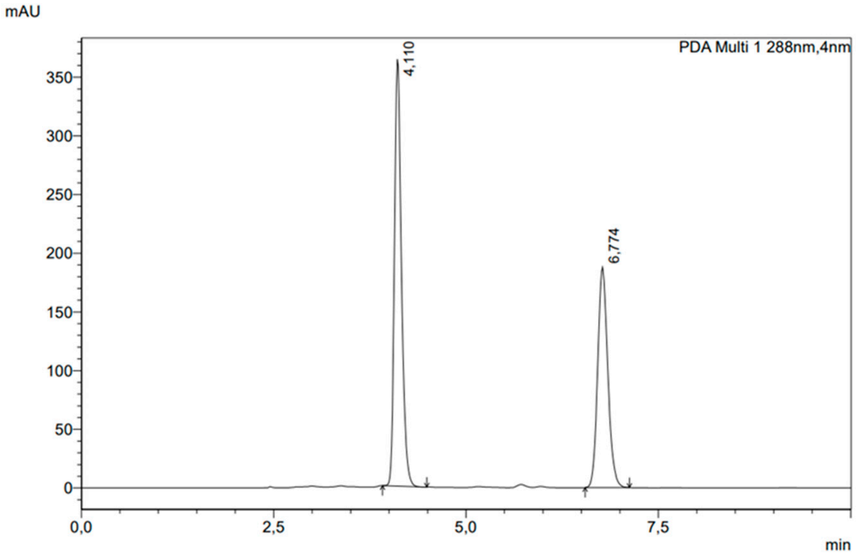

(a)

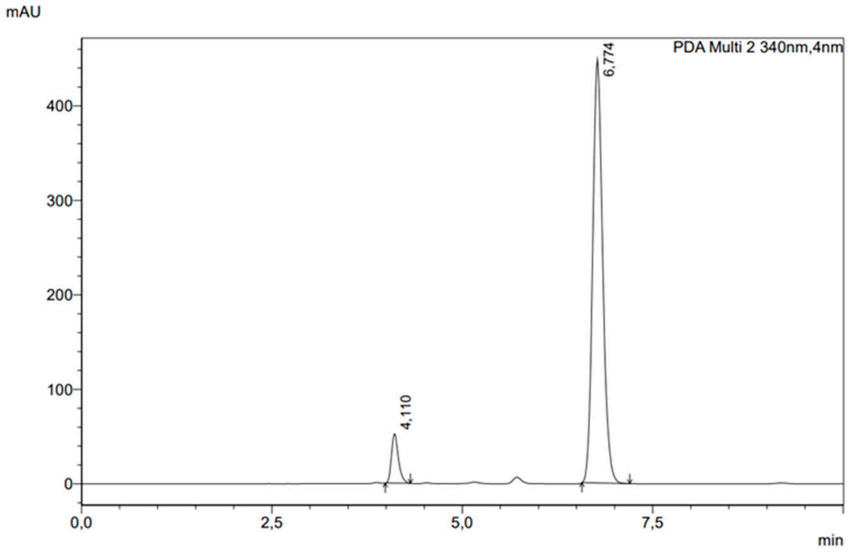

(b)

Figure S3. Chromatograms of hesperetin (**a**) and piperine (**b**).

Chromatographic conditions

- Stationary phase: Dr. Maisch ReproSil-Pur Basic-C18 100 Å column, 5 µm particle size, 250 × 4.60 mm
- Mobile phase: methanol/0.1% acetic acid (80:20 v/v)
- Column temperature: 30 °C
- Flow rate: 1.0 mL·min^{-1}

Table S1. Validation parameters of HPLC-DAD method for concentration determination of hesperetin and piperine

**Hesperetin**	
Parameter	Hesperetin dissolved in 50% DMSO; Injection volume 10 µl
Linearity range (mg·mL^{-1})	0.00008 – 0.48
Correlation coefficient (r)	0.9999
a ± S_a	38446721 ± 196766
b ± S_b	insignificant (α=0.05)
LOD (mg·mL^{-1})	0.0075
LOQ (mg·mL^{-1})	0.0228
Retention Time	4.11
**Piperine**	
Parameter	Piperine dissolved in 50% DMSO; Injection volume 10 µl
Linearity range (mg·mL^{-1})	0.00008 – 0.16
Correlation coefficient (r)	0.9975
a ± S_a	68194881 ± 1815271

$b \pm S_b$	insignificant (α=0.05)
LOD ($mg \cdot mL^{-1}$)	0.0136
LOQ ($mg \cdot mL^{-1}$)	0.0411
Retention Time (min)	6.77

References

24. Garrido, B.; González, S.; Hermosilla, J.; Millao, S.; Quilaqueo, M.; Guineo, J.; Acevedo, F.; Pesenti, H.; Rolleri, A.; Shene, C. Carbonate-β-cyclodextrin-based nanosponge as a nanoencapsulation system for piperine: physicochemical characterization. *J. Soil Sci. Plant Nutr.* **2019**, *19*, 620–630.
51. Guo, J.; Tang, W.; Lu, S.; Fang, Z.; Tu, K.; Zheng, M. Solubility improvement of hesperetin by using different octenyl succinic anhydride modified starches. *LWT* **2018**, *95*, 255–261.
52. Krysa, M.; Szymańska-Chargot, M.; Zdunek, A. FT-IR and FT-Raman fingerprints of flavonoids–a review. *Food Chem.* **2022**, 133430.
53. Gorgani, L.; Mohammadi, M.; Najafpour, G.D.; Nikzad, M. Sequential microwave-ultrasound-assisted extraction for isolation of piperine from black pepper (Piper nigrum L.). *Food Bioprocess Technol.* **2017**, *10*, 2199–2207.
54. Quilaqueo, M.; Millao, S.; Luzardo-Ocampo, I.; Campos-Vega, R.; Acevedo, F.; Shene, C.; Rubilar, M. Inclusion of piperine in β-cyclodextrin complexes improves their bioaccessibility and in vitro antioxidant capacity. *Food Hydrocoll.* **2019**, *91*, 143–152.
55. Školáková, T.; Slámová, M.; Školáková, A.; Kadeřábková, A.; Patera, J.; Zámostný, P. Investigation of dissolution mechanism and release kinetics of poorly water-soluble tadalafil from amorphous solid dispersions prepared by various methods. *Pharmaceutics* **2019**, *11*, 383.
56. Ijaz, Q.A.; Latif, S.; Rashid, M.; Arshad, M.S.; Hussain, A.; Bukhari, N.I.; Riaz, S.; Abbas, N. Preparation and Characterization of pH-Independent Sustained-Release Tablets Containing Hot Melt Extruded Solid Dispersions of Clarithromycin. *AAPS PharmSciTech* **2021**, *22*, 1–12.
57. Fu, Q.; Fang, M.; Hou, Y.; Yang, W.; Shao, J.; Guo, M.; Li, M.; Li, J.; Wang, Y.; He, Z. A physically stabilized amorphous solid dispersion of nisoldipine obtained by hot melt extrusion. *Powder Technol.* **2016**, *301*, 342–348.
58. Liu, K.; Liu, H.; Li, Z.; Li, W.; Li, L. In vitro dissolution study on inclusion complex of piperine with ethylenediamine-β-cyclodextrin. *J. Incl. Phenom. Macrocycl. Chem.* **2020**, *96*, 233–243.
